# Effect of transcutaneous electrical acupoint stimulation on perioperative hypothermia in video-assisted thoracoscopic surgery: a randomized controlled trial

**DOI:** 10.3389/fmed.2026.1799152

**Published:** 2026-04-30

**Authors:** Yajie Zhao, Hansheng Liang, Kaidi Kang, Menghan Su, Qian Gao, Yaqing Wu, Weixin Zhang, Yue Li, Liang Sun, Yi Feng

**Affiliations:** 1 Department of Anesthesiology, Peking University People’s Hospital, Beijing, China; 2 Department of Anesthesiology, Peking University People's Hospital Shijiazhuang Campus, Shijiazhuang People's Hospital, Shijiazhuang, China

**Keywords:** acupuncture, complications, general anesthesia, perioperative hypothermia, transcutaneous electrical acupoint stimulation, video-assisted thoracoscopic surgery

## Abstract

**Background:**

Perioperative hypothermia is common during video-assisted thoracoscopic surgery (VATS) and is associated with adverse clinical outcomes. Transcutaneous electrical acupoint stimulation (TEAS) is a noninvasive neuromodulation technique that may modulate thermoregulation; however, evidence from randomized controlled trials remains limited. This study evaluated whether TEAS prevents perioperative hypothermia in patients undergoing VATS.

**Methods:**

Adults scheduled for elective VATS under general anesthesia were randomized (1:1) to receive TEAS at predefined acupoints from 30 min before anesthesia induction until the end of surgery, or sham stimulation. The primary outcome was the incidence of perioperative hypothermia, defined as a core temperature <36 °C. Secondary outcomes included intraoperative temperature trajectories, hemodynamic parameters, postoperative recovery metrics, and complications.

**Results:**

Of 92 enrolled patients, 88 completed the trial (mean age, 52.8 ± 10.0 years; 41 women) and were included in the final analysis (44 per group). The incidence of perioperative hypothermia was lower in the TEAS group than in the control group (15.9% [7/44] vs. 40.9% [18/44]; *p* = 0.009; OR, 0.273; 95% CI, 0.100–0.748). Patients receiving TEAS maintained higher intraoperative core temperatures. The incidence of postoperative complications was lower in the TEAS group than in the control group (2.3% [1/44] vs. 18.2% [8/44]; *p* = 0.030; OR, 0.105; 95% CI, 0.012–0.877). However, these findings were primarily driven by differences in postoperative shivering. No between-group differences were observed in other secondary outcomes. No TEAS-related adverse events were observed.

**Conclusion:**

Perioperative TEAS improved thermoregulation, reduced the incidence of perioperative hypothermia and shivering in VATS. As a safe, noninvasive intervention, TEAS may be a useful adjunct to standard perioperative temperature management.

**Clinical trial registration:**

https://itmctr.ccebtcm.org.cn/mgt/project/view/-7254737145961978300, Identifier ITMCTR2024000425.

## 1 Introduction

Perioperative hypothermia, defined as an unintended decrease in core temperature to <36 °C during surgery, is a key quality metric in anesthetic care. Reported incidence rates range from 7 to 90%, and perioperative hypothermia is associated with clinically important complications, including coagulopathy, surgical site infections, and cardiovascular morbidity (1–3). In recognition of its relevance to enhanced recovery after surgery (ERAS), the National Center for Quality Control of Anesthesia Specialties incorporated temperature management into anesthesia quality assessment protocols in 2022 (4). Although video-assisted thoracoscopic surgery (VATS) is minimally invasive, patients remain vulnerable to substantial heat loss via three main mechanisms: (1) prolonged exposure of the thoracic cavity, (2) convective heat loss driven by CO₂ insufflation during pneumothorax, and (3) heat extraction by suction devices. In our previous study, the incidence of hypothermia in this population was 72% (5), and rates up to 93% have been reported in robotic-assisted thoracoscopic procedures (6). These observations highlight persistent limitations of current multimodal warming strategies in this setting.

Acupuncture is one of the most widely used complementary and alternative medicine (CAM) modalities worldwide. Current stimulation techniques include manual acupuncture, electroacupuncture, and transcutaneous electrical acupoint stimulation (TEAS). TEAS is a noninvasive neuromodulation technique that delivers electrical stimulation to specific acupoints, allowing precise control of stimulation parameters. TEAS has attracted attention in perioperative medicine because it offers several potential benefits, including reduced anesthetic requirements, less postoperative pain (7–9), lower rates of postoperative nausea and vomiting (PONV) (10, 11) and postoperative sleep disturbances (POSD) (12, 13), and improved postoperative recovery (4, 14). Preliminary studies suggest that acupuncture-related interventions may reduce perioperative hypothermia and postanesthetic shivering (9, 15, 16). However, high-quality randomized controlled trials evaluating TEAS for perioperative temperature management in thoracoscopic surgery are lacking.

Mechanistically, stimulation of selected acupoints can modulate autonomic nervous system activity by altering sympathetic outflow and peripheral vasomotor tone. Such modulation may enhance thermoregulation and help maintain core temperature stability during anesthesia (17–21). The acupoints GV14, GV4, CV4, and KI1 were selected based on traditional Chinese medicine (TCM) principles aimed at warming Yang, tonifying Kidney function, and restoring systemic energy balance, all of which are considered relevant to preventing perioperative hypothermia. Therefore, we hypothesized that perioperative TEAS delivered at four predefined acupoints (GV14, GV4, CV4, and KI1) on the operative side would reduce the incidence of hypothermia and improve postoperative recovery in patients undergoing VATS.

## 2 Materials and methods

### 2.1 Design, setting, and ethics

**Figure 1:**
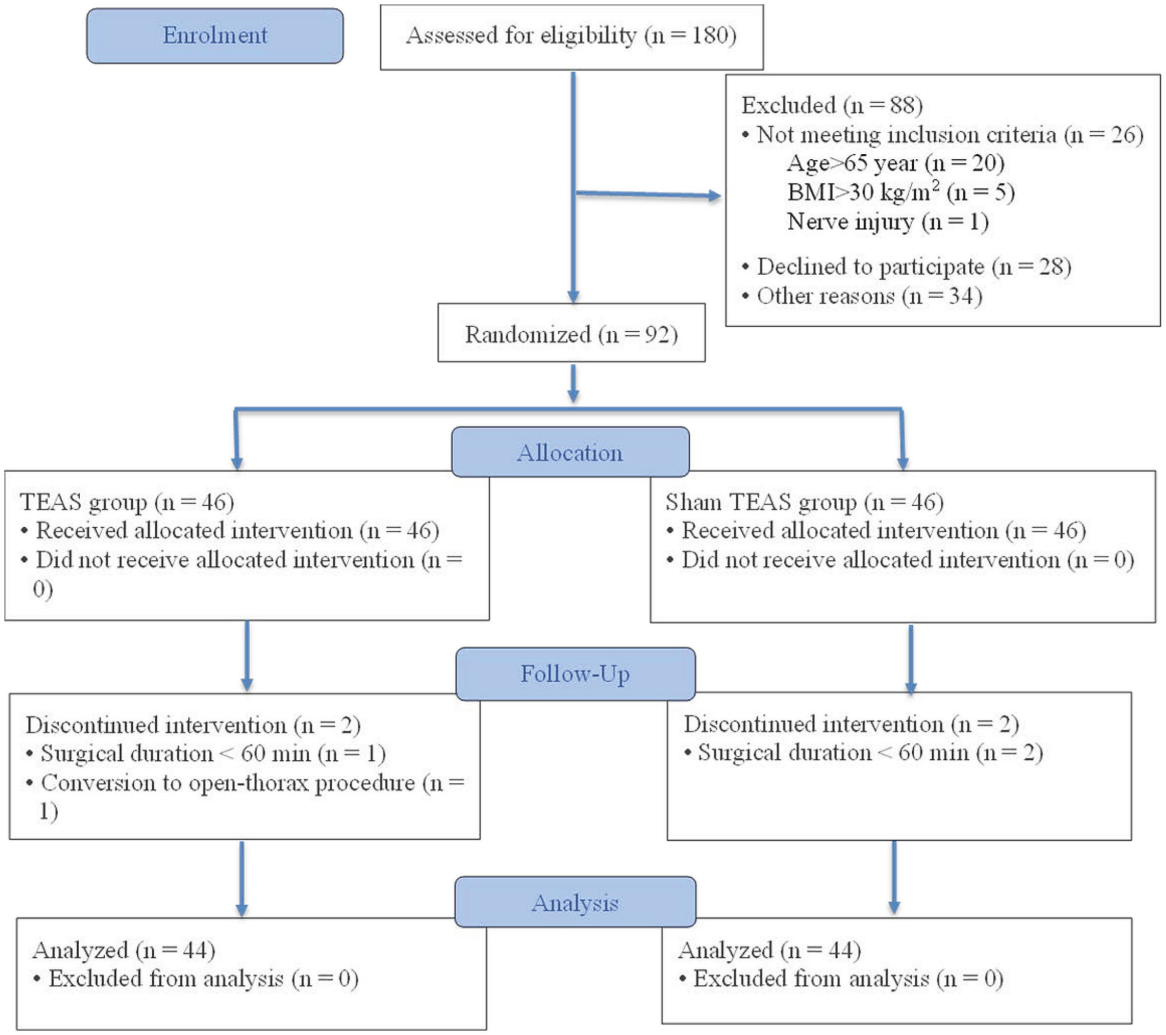
Flow diagram of patient enrollment. BMI, body mass index; TEAS, transcutaneous electrical acupoint stimulation.

This randomized controlled trial was conducted at Peking University People’s Hospital between October 2024 and March 2025 and was approved by the Ethics Committee of Peking University People’s Hospital (Approval No. 2024PHB177-001). Written informed consent was obtained from all participants before enrollment. The study was conducted in accordance with the Declaration of Helsinki. The trial was registered with the International Traditional Medicine Clinical Trial Registry (ITMCTR2024000425; August 12, 2024). The CONSORT flow diagram is shown in Figure 1.

### 2.2 Participants

This study was conducted at Peking University People’s Hospital in Beijing, China. The inclusion criteria were as follows: (1) patients scheduled for elective VATS (lobectomy or segmentectomy); (2) age 18–65 years; and (3) American Society of Anesthesiologists (ASA) physical status I–II. Patients meeting any of the following criteria were excluded: (1) cardiac pacemakers or other electronic implants; (2) diabetes mellitus, Raynaud’s syndrome, or other thermoregulatory disorders; (3) a history of febrile illness or use of medications affecting thermoregulation within 30 days; (4) refusal to participate; (5) conversion to an open thoracotomy; or (6) surgical duration <60 min.

### 2.3 Randomization and blinding

The randomization sequence was generated using IBM SPSS Statistics (version 27.0) with simple randomization by an independent staff member not involved in the trial. Allocation concealment was ensured using sealed, opaque envelopes. Participants were randomized in a 1:1 ratio to receive TEAS or sham stimulation. Participants were informed that electrical stimulation might or might not be perceptible. Outcome assessors, anesthesiologists responsible for intraoperative temperature monitoring, and data analysts were blinded to group assignment. Treatment providers were not blinded because they administered the interventions. Communication between statisticians, outcome assessors, and clinical staff was restricted to minimize the risk of unblinding. Due to the nature of the intervention, sensory perception during stimulation may have differed between the TEAS and sham groups. Formal post-intervention assessment of blinding effectiveness was not conducted. However, strict blinding of all personnel involved in outcome measurement and data analysis was maintained to reduce detection bias. Importantly, the primary outcome was objective core temperature measurements, which may reduce the impact of subjective bias on the main results.

### 2.4 Interventions and control

To minimize intraoperative heat loss, all patients were transferred to a preoperative preparation room equipped with heated mattresses and blankets set to 38 °C. Standardized thermal protection measures were implemented in both groups: circulating blankets (43 ± 0.5 °C) were activated 10 min before transfer to the operating room; prewarmed surgical drapes (22 ± 0.5 °C) covered all nonoperative areas; and intravenous fluids were preheated to 38 ± 0.5 °C. The operating room temperature was maintained at 22 ± 0.5 °C in accordance with standard institutional practice for thoracic surgery.

**Figure 2:**
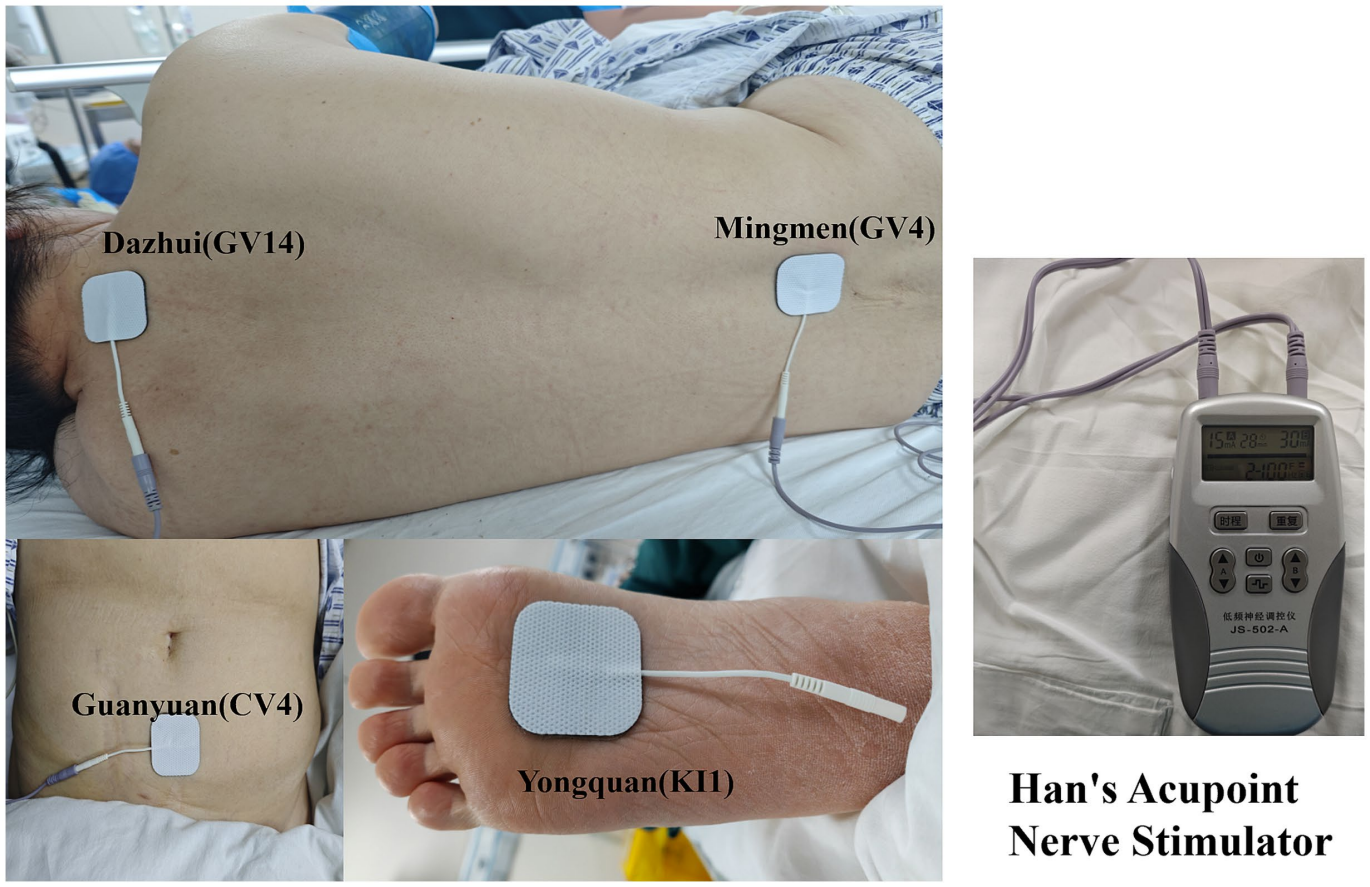
Photographs showing the placement of stimulation pads on Mingmen (GV4), Dazhui (GV14), Guanyuan (CV4), Yongquan (KI1), and Han’s acupoint nerve stimulator.

The standardized TEAS protocol was as follows: upon arrival in the preoperative preparation room, electrode pads (approximately 40 mm in diameter; S4040, Shenping Xintai Medical Technology, Wuxi, China) were placed at the GV14, GV4, CV4, and KI1 acupoints on the operative side (Figure 2). Participants in the TEAS group received transcutaneous electrical stimulation using a Han’s Acupoint Nerve Stimulator (HANS-200A; Jisheng Co., Nanjing, China) with alternating dense–disperse waves (2/100 Hz) and an intensity of 15–30 mA, adjusted to the individual sensory threshold. TEAS was delivered from 30 min before anesthesia induction until the end of surgery. In the sham TEAS group, electrodes were placed at the same acupoints, but no electrical stimulation was delivered. In the TEAS group, stimulation was immediately discontinued if the core temperature exceeded 37.0 °C. Patient tolerance to stimulation was monitored throughout the intervention period. The procedure was terminated immediately in case of poor tolerance and discomfort. All procedures were performed by trained clinicians with at least 5 years of clinical experience.

For all patients, forced-air warming (FAW) systems (Bair Hugger™, 3 M) were activated as a rescue intervention when core temperature fell below 35.5 °C. The study protocol was developed based on our current clinical practice, given the limited numbers of FAW systems. Additionally, this strategy was implemented with a focus on low-carbon, energy-efficient practices, prioritizing reusable warming modalities over the routine use of FAW systems.

### 2.5 Standard procedure for anesthesia and analgesia

Anesthesia was induced with intravenous midazolam (0.05 mg/kg), etomidate (0.2 mg/kg), sufentanil (0.5 μg/kg), and cisatracurium (0.2 mg/kg). A double-lumen endotracheal tube was inserted under direct laryngoscopy, and its position was confirmed by auscultation and fiberoptic bronchoscopy. After intubation, mechanical ventilation was set to a tidal volume of 6–8 mL/kg (predicted body weight), a respiratory rate of 10–15 breaths/min, an inspiratory-to-expiratory ratio of 1:2, PEEP of 5 cm H_2_O, and a PETCO_2_ target of 30–35 mmHg (4.0–4.7 kPa). Anesthesia was maintained with total intravenous anesthesia (TIVA) using propofol (4–6 mg/kg/h), remifentanil (0.1–0.3 μg/kg/min), and dexmedetomidine (0.2–0.7 μg/kg/h), with the bispectral index maintained at 40–60.

For postoperative pain management, all patients received multimodal analgesia. Ultrasound-guided thoracic paravertebral block (TPVB; 0.4% ropivacaine, 30 mL) and serratus anterior plane block (0.4% ropivacaine, 10 mL) were performed preoperatively. Preoperative TPVB may provide better protection against perioperative hypothermia than postoperative TPVB (22). Oxycodone and a disposable flurbiprofen axetil pump were used for patient-controlled intravenous analgesia (PCIA). Tracheal extubation was performed in the post-anesthesia care unit (PACU) by an attending anesthesiologist.

### 2.6 Outcomes

Core temperature was continuously measured using tympanic membrane temperature with a calibrated infrared tympanic thermometer (ThermoScan™ 7 IRT 6520; Braun, Kronberg, Germany) at predefined perioperative time points: T0, hospital admission (baseline); T1–T2, admission to and discharge from the preoperative preparation room; T3, operating room admission; T4, immediately after anesthesia induction; T5, 30 min; T6, 60 min; T7, 90 min after induction; T8, skin closure; T9–T10, PACU admission and discharge; and T11–T13, daily measurements on postoperative days 1–3.

The primary outcome was the incidence of perioperative hypothermia, defined as recorded core temperature measurement <36 °C at the predefined time points, regardless of duration.

Esophageal or nasopharyngeal temperature probes provide highly accurate measurements of core temperature, but they are not practical for routine use in conscious patients. Some studies have demonstrated that averaged infrared tympanic thermometers are sufficiently reliable for detecting significant perioperative hypothermia and monitoring temperature trends (23). However, infrared tympanic thermometry may produce lower readings compared to invasive core temperature measurements, and the lateral decubitus position during VATS may introduce additional measurement variability. Consequently, it is important to consider these limitations when interpreting our results. To enhance measurement accuracy and minimize random variability, three consecutive readings were obtained at each time point and averaged for analysis. All measurements were taken using the same device and standardized method by trained staff throughout the study.

Secondary outcomes included core temperature trajectories, hemodynamic parameters, postoperative recovery metrics, and complications. Hemodynamic parameters included mean arterial pressure (MAP) and heart rate (HR), measured at time points T0–T13. Postoperative recovery metrics included extubation time (from awakening to tracheal extubation) and length of hospital stay. The incidence and severity of postoperative complications were assessed using the Clavien–Dindo grading system on postoperative days 1–7 (24).

### 2.7 Sample size calculation

The sample size was calculated based on a pilot study involving 40 patients undergoing video-assisted thoracoscopic lobectomy. In the pilot study, perioperative hypothermia occurred in 65% of patients in the sham TEAS group and 30% in the TEAS group. With 80% power (1 − *β*) and a two-sided *α* of 0.05, the minimum required sample size was 40 patients per group. To account for a 5% dropout rate, the sample size was increased to 43 patients per group. In total, 92 participants were randomized to account for potential intraoperative exclusions; 88 (44 per group) completed the trial, meeting the prespecified sample size requirement. This sample size was calculated exclusively for the primary outcome of perioperative hypothermia.

### 2.8 Statistical analysis

Statistical analyses were performed using IBM SPSS Statistics (version 27.0; Armonk, NY, United States). Baseline characteristics are presented as the mean ± standard deviation (SD) for normally distributed continuous variables, the median (interquartile range [IQR]) for skewed variables, and numbers (percentages) for categorical variables. Normality was assessed using the Shapiro–Wilk test. Independent two-sample *t* tests were used for normally distributed continuous variables, whereas nonparametric tests (Mann–Whitney U test) were used for skewed data. Categorical variables were compared using Pearson’s chi-square (χ^2^) test or Fisher’s exact test, as appropriate. For repeated measurements, repeated-measures analysis of variance (ANOVA) was used to compare groups over time. For non-normally distributed repeated measurements, the Mann–Whitney U test was used at each time point, with the Benjamini–Hochberg procedure applied to control for multiple comparisons. A two-sided *p* value <0.05 was considered statistically significant.

## 3 Results

### 3.1 Patient characteristics

Between October 15, 2024, and March 1, 2025, 180 patients were screened, and 88 were excluded for not meeting the eligibility criteria. A total of 92 participants were randomized to the TEAS group (*n* = 46) or the sham TEAS group (*n* = 46). In the TEAS group, two participants were excluded from the analysis because of conversion to open thoracotomy (*n* = 1) or a surgical duration <60 min (*n* = 1). In the sham TEAS group, two participants were excluded because of a surgical duration <60 min. Ultimately, 88 participants were included in the final analysis (44 per group) (Figure 1). Baseline characteristics were comparable between the two groups (all *p* > 0.05) (Table 1). There were no significant between-group differences in anesthesia duration, operative time, intraoperative fluid volume, urine output, or blood loss (all *p* > 0.05) (Table 1).

**Table 1:** Baseline characteristics of the patients.

Characteristics	TEAS (*n* = 44)	Sham control (*n* = 44)
Age, year	51.1 ± 9.4	54.5 ± 10.5
Sex (male/female)	23/21	24/20
BMI, kg/m^2^	24.1 ± 3.3	25.0 ± 4.6
ASA grading, I/II	1/43	2/42
Anesthesia duration, min	154.0 (135.0, 184.7)	155.5 (135.7, 182.2)
Operation time, min	90.0 (65.0, 114.2)	85.0 (72.5, 112.2)
Fluid volume, ml	700.0 (600.0, 800.0)	800.0 (700.0, 875.0)
Urine output, ml	400.0 (200.0, 575.0)	300.0 (102.5, 500.0)
Blood loss, ml	20.0 (10.0, 20.0)	20.0 (10.0, 37.5)

Data were presented as mean ± SD, M (IQR), and number of patients. SD, standard deviation; M, median; IQR, interquartile range; BMI, body mass index; ASA, American Society of Anesthesiologists; TEAS, transcutaneous electrical acupoint stimulation.

### 3.2 Primary outcome

The incidence of perioperative hypothermia was significantly lower in the TEAS group than in the sham group (15.9% [7/44] vs. 40.9% [18/44]; *p* = 0.009; OR, 0.273; 95% CI, 0.100–0.748). Time-dependent analysis demonstrated a sustained reduction in hypothermia incidence in the TEAS group, particularly during the intraoperative period (T6–T9). The proportion of patients requiring rescue active warming did not differ between the two groups (Tables 2, 3).

**Table 2:** Comparison of hypothermia, active warming intervention, postoperative complication, and recovery parameters between the two groups.

Outcomes	TEAS (*n* = 44)	Sham control (*n* = 44)	OR (95% CI)	MD (95% CI)	*p*-value
Perioperative hypothermia, *n* (%)	7 (15.9)*	18 (40.9)	0.273 (0.100 to 0.748)		0.009
Active warming intervention, *n* (%)	2 (4.5)	4 (9.1)	0.476 (0.083 to 2.745)		0.676
^†^Postoperative complication, *n* (%)	1 (2.3)*	8 (18.2)	0.105 (0.012 to 0.877)		0.030
Extubation time, min	12.7 ± 5.9	14.0 ± 6.2		1.35 (−1.29 to 3.98)	0.313
Hospital stays, days	7.9 ± 2.7	7.8 ± 2.5		−0.09 (−1.20 to 1.02)	0.871

Data were presented as *n* (%), mean ± standard deviation. * *P* < 0.05 vs. Sham TEAS group. ^†^Postoperative complications included postoperative infection (*n* = 1) in the TEAS group; postoperative infections (*n* = 2), atrial fibrillation (*n* = 1), and postoperative shivering (*n* = 5) in the sham TEAS group. OR, odds ratio; MD, mean difference; CI, confidence intervals; TEAS, transcutaneous electrical acupoint stimulation.

**Table 3:** Point-prevalence of hypothermia between the two groups at each time point.

Time point	TEAS (*n* = 44), *n* (%)	Sham control (*n* = 44), *n* (%)	*P*-value
T0–T4	0 (0)	0 (0)	–
T5	1 (2.3)	2 (4.5)	0.556
T6	3 (6.8)	10 (22.7)	0.030*
T7	7 (15.9)	18 (40.9)	0.009*
T8	4 (9.0)	16 (36.4)	0.003*
T9	4 (9.0)	16 (36.4)	0.003*
T10	0 (0)	2 (4.5)	0.152
T11–13	0 (0)	0 (0)	–

Data were presented as *n* (%). * *P* < 0.05 vs. Sham TEAS group. T0: Hospital admission. T1: Preoperative preparation room admission. T2: Preoperative preparation room exit. T3: Operating room admission. T4: Immediately post-anesthesia induction. T5: 30 min after induction. T6: 60 min after induction. T7: 90 min after induction. T8: Skin closure. T9: PACU admission. T10: PACU discharge. T11: Postoperative day 1. T12: Postoperative day 2. T13: Postoperative day 3. PH, perioperative hypothermia; TEAS, transcutaneous electrical acupoint stimulation; PACU, post-anesthesia care unit.

### 3.3 Secondary outcomes

#### 3.3.1 Core body temperature trends

**Figure 3:**
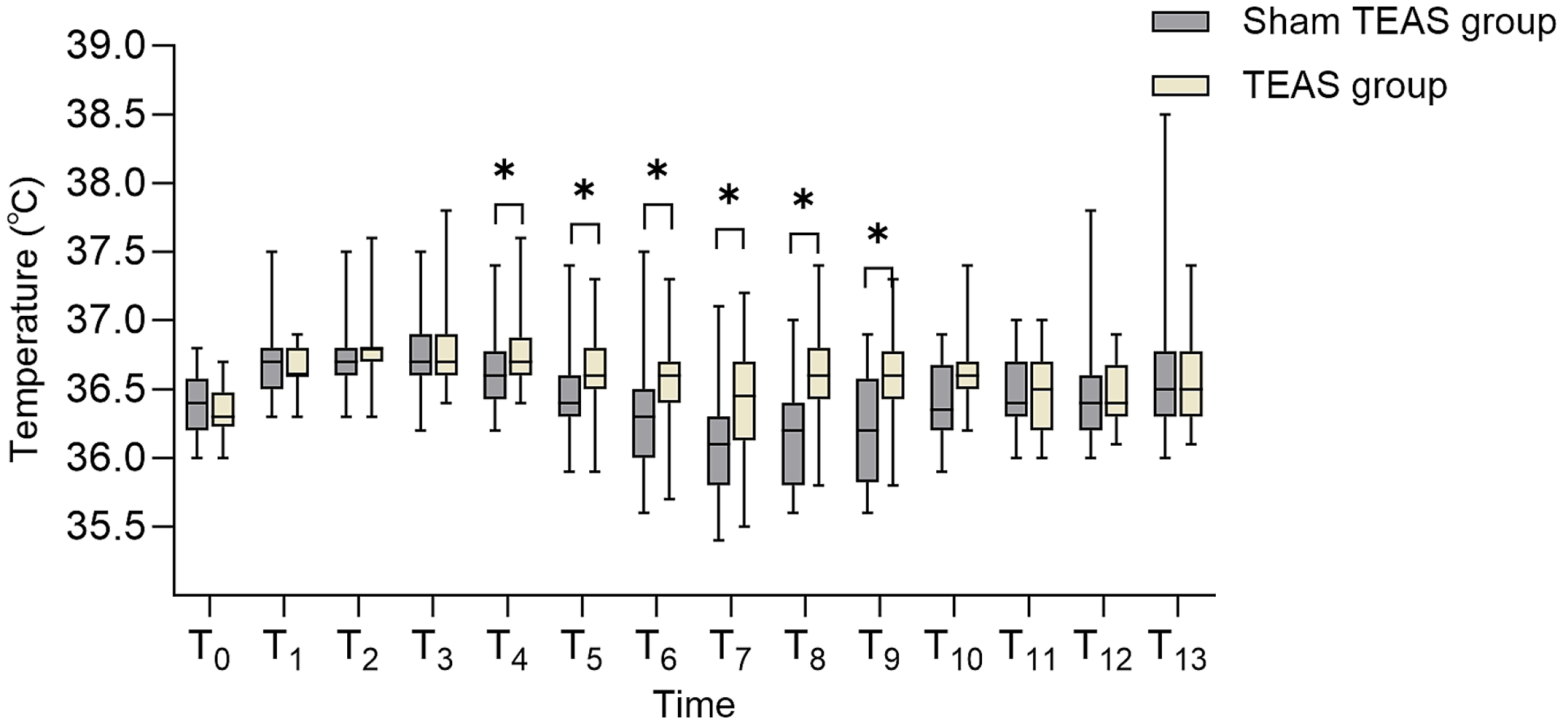
Core body temperature profiles at different phases. * *P* Ben_jamini-Hochberg_ <0.05 vs. sham TEAS group. T0: Hospital admission. T1: Preoperative preparation room admission. T2: Preoperative preparation room exit. T3: Operating room admission. T4: Immediately post-anesthesia induction. T5: 30 min after induction. T6: 60 min after induction. T7: 90 min after induction. T8: Skin closure. T9: PACU admission. T10: PACU discharge. T11: Postoperative day 1. T12: Postoperative day 2. T13: Postoperative day 3. PACU, post-anesthesia care unit; TEAS, transcutaneous electrical acupoint stimulation.

Baseline core temperatures did not differ between the TEAS and sham TEAS groups at hospital admission (T0, 36.3 °C [IQR, 36.2–36.5 °C] vs. 36.4 °C [IQR, 36.2–36.6 °C]; *p* = 0.106) or at T1, T2, and T3 (all *p* > 0.05). Compared with the sham TEAS group, the TEAS group maintained higher core temperatures during key perioperative time points from induction to early recovery (T4–T9; P_Benjamini–Hochberg_ <0.05). No between-group differences were observed on postoperative days 1–3 (all *p* > 0.05) (Table 3; Figure 3).

#### 3.3.2 Hemodynamic parameters

**Figure 4:**
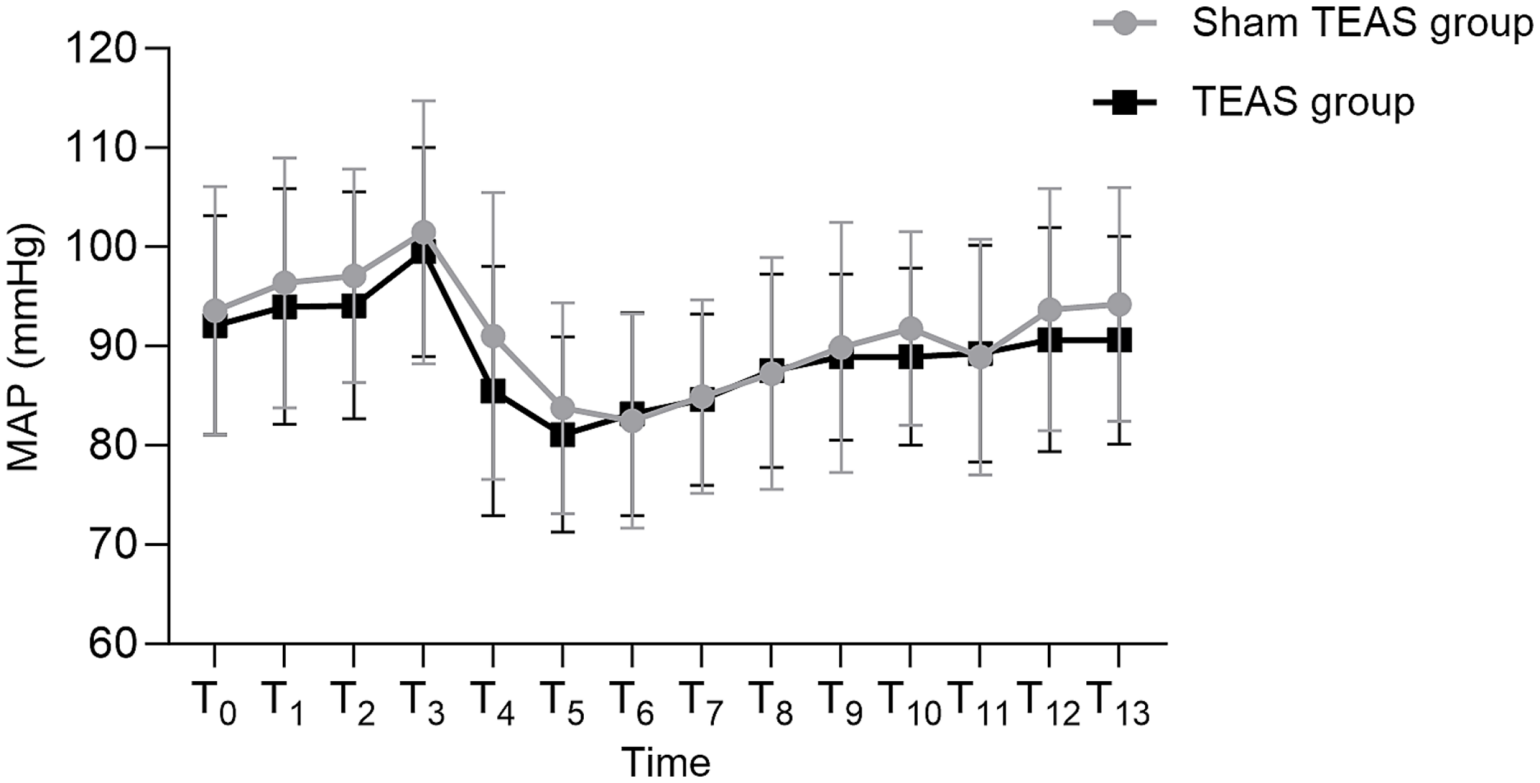
Comparison of MAP at different time points between the two groups. T0: Hospital admission. T1: Preoperative preparation room admission. T2: Preoperative preparation room exit. T3: Operating room admission. T4: Immediately post-anesthesia induction. T5: 30 min after induction. T6: 60 min after induction. T7: 90 min after induction. T8: Skin closure. T9: PACU admission. T10: PACU discharge. T11: Postoperative day 1. T12: Postoperative day 2. T13: Postoperative day 3. MAP, mean arterial pressure; HR, heart rate; PACU, post-anesthesia care unit; TEAS, transcutaneous electrical acupoint stimulation.

**Figure 5:**
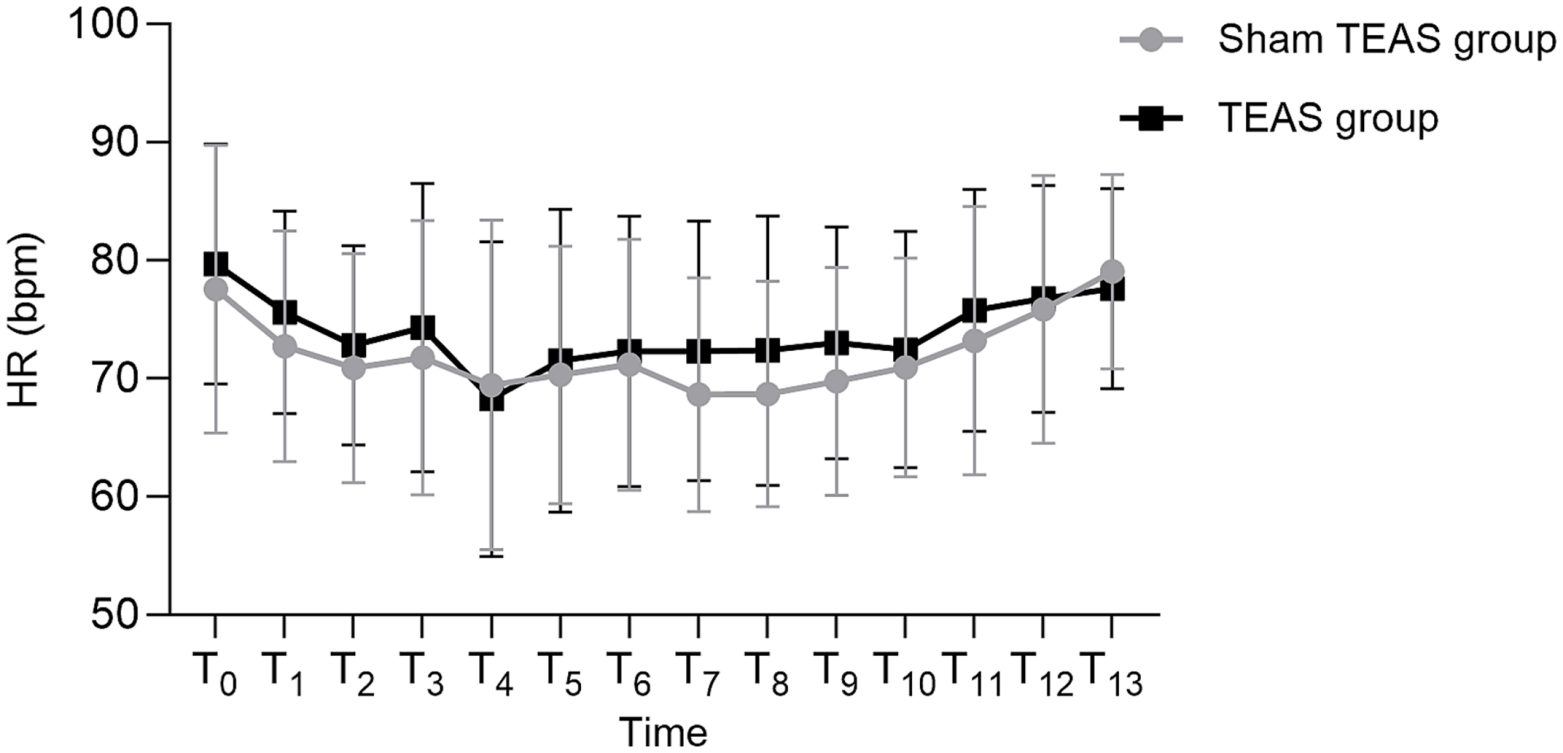
Comparison of HR at different time points between the two groups. T0: Hospital admission. T1: Preoperative preparation room admission. T2: Preoperative preparation room exit. T3: Operating room admission. T4: Immediately post-anesthesia induction. T5: 30 min after induction. T6: 60 min after induction. T7: 90 min after induction. T8: Skin closure. T9: PACU admission. T10: PACU discharge. T11: Postoperative day 1. T12: Postoperative day 2. T13: Postoperative day 3. MAP: Mean arterial pressure. HR: Heart rate. PACU: Post-anesthesia care unit. TEAS: Transcutaneous electrical acupoint stimulation.

No between-group differences were observed in mean arterial pressure (MAP) or heart rate (HR) at any time point (all *p* > 0.05) (Figures 4, 5).

#### 3.3.3 Postoperative recovery metrics

Extubation time (12.7 ± 5.9 min vs. 14.0 ± 6.2 min; *p* = 0.313; mean difference, 1.35 min; 95% CI, −1.29 to 3.98) and length of hospital stay (7.9 ± 2.7 days vs. 7.8 ± 2.5 days; *p* = 0.871; mean difference, −0.09 days; 95% CI, −1.20 to 1.02) did not differ between the TEAS and sham TEAS groups (Table 2).

#### 3.3.4 Complications

The incidence of postoperative complications was lower in the TEAS group than in the sham TEAS group (2.3% [1/44] vs. 18.2% [8/44]; *p* = 0.030; OR, 0.105; 95% CI, 0.012–0.877). All postoperative complications were graded as Clavien–Dindo grade I or II. Postoperative infection occurred in one patient in the TEAS group and in two patients in the sham TEAS group. Additional complications in the sham TEAS group included atrial fibrillation (*n* = 1) and postoperative shivering (*n* = 5). When analyzed individually, only postoperative shivering differed significantly between groups, whereas no significant differences were observed for infection or atrial fibrillation (Table 2).

#### 3.3.5 Safety of TEAS

No TEAS-related adverse events, including pain, erythema, or swelling, were reported.

## 4 Discussion

This study showed that TEAS delivered at specific acupoints reduced the incidence of perioperative hypothermia during VATS (15.9% vs. 40.9%; *p* = 0.009). Patients in the TEAS group maintained higher core temperatures during surgery (0, 30, 60, and 90 min after induction and at skin closure; all *p* < 0.05), suggesting that TEAS may enhance thermoregulation rather than simply reduce heat loss. The observed relative reduction in hypothermia incidence, corresponding to a substantial effect size (OR, 0.273), is consistent with, and extends, previous work on acupuncture-related thermoregulation (16). Earlier studies primarily focused on prewarming or postoperative thermal comfort (16, 25), whereas our findings suggest that continuous intraoperative TEAS may modulate physiological pathways involved in heat production and redistribution.

The selection of GV14, GV4, CV4, and KI1 was based on both TCM theory and existing research. In TCM, perioperative hypothermia is commonly attributed to “Yang deficiency” and impaired warming function (17). GV14 is considered to regulate systemic Yang-Qi as the meeting point of all Yang meridians. GV4 and CV4 are key points for tonifying Kidney Yang and have been shown to improve tissue oxygenation and energy metabolism (26), thereby improving Yang deficiency. KI1, located on the Kidney meridian, is traditionally used to harmonize Yin–Yang balance and facilitate the distribution of Qi (21). This combined strategy integrates Kidney meridian tonification with Yang Qi activation to achieve a synergistic effect that may help maintain thermal homeostasis.

Acupuncture may influence thermoregulation through central, peripheral, and metabolic pathways. Centrally, the hypothalamic preoptic area and the hypothalamus–periaqueductal gray (PAG)–cortical network may be involved, potentially modulating sympathetic outflow, neurotransmitter release, and counteracting anesthesia-related thermoregulatory dysfunction (27–31). Peripherally, TEAS may attenuate anesthetic-induced vasodilation and improve peripheral heat distribution (8, 32–35). Metabolically, electroacupuncture has been associated with activation of the peroxisome proliferator-activated receptor γ coactivator 1α (PGC-1α)–irisin–uncoupling protein 1 (UCP1) pathway and central glucagon-like peptide-1 (GLP-1) neurons, which could facilitate non-shivering thermogenesis and improve metabolic efficiency (33, 36), thereby contributing to perioperative temperature stability. While multiple central, peripheral, and metabolic pathways may contribute to the thermoregulatory effects of TEAS, the present study was not designed to investigate these mechanisms. Future research incorporating measures of autonomic activity, vascular responses, or relevant biomarkers is warranted to further elucidate the underlying biological processes.

Our findings are consistent with previous reports that perioperative hypothermia is associated with postoperative shivering, infection, arrhythmia, and delayed recovery (2, 6). The TEAS group had fewer postoperative complications (2.3% [1/44] vs. 18.2% [8/44]; *p* = 0.030). When analyzed individually, postoperative shivering was the only complication that differed significantly between groups, while other complications, such as infection and atrial fibrillation, were infrequent and showed no meaningful differences. Since shivering is a direct thermoregulatory response to perioperative hypothermia, its reduction aligns with the improved thermal stability observed in the TEAS group. However, considering the differing pathophysiological mechanisms underlying these complications, the overall clinical benefit observed may be overestimated.

Although extubation time and length of hospital stay tended to be shorter in the TEAS group, these differences were not statistically significant, possibly due to limited statistical power and the relatively fast perioperative turnover in our institution. Previous studies have suggested that TEAS may exert broader physiological effects, including modulation of inflammatory responses, attenuation of oxidative stress, and potential neuroprotective properties (9, 34, 35, 37). However, no mechanistic biomarkers were assessed in the present study, and therefore the clinical effect of TEAS on postoperative complications remains speculative and requires further validation. Furthermore, the analysis of postoperative shivering was affected by sparse data, with zero events in the TEAS group requiring continuity correction and yielding a wide confidence interval. These factors indicate substantial statistical uncertainty.

Taken together, the findings suggest a coherent mechanism in which TEAS may improve perioperative thermoregulation, thereby reducing hypothermia and its immediate consequence, postoperative shivering.

The operating room temperature is an important factor influencing hypothermia during general anesthesia (38). In clinical practice, the temperature should be dynamically adjusted based on specific circumstances. Notably, evidence from a randomized factorial trial suggests that ambient temperature has minimal effect on core temperature when active warming is used (39). In the present study, all patients were provided with warming blankets. Therefore, the operating room temperature can be set at 22 °C to balance surgical comfort, infection control, and anesthesia-related requirements.

Previous studies suggest that acupuncture may increase cardiac output and stroke volume and stabilize hemodynamic parameters, such as mean arterial pressure (MAP) and heart rate (HR) (40, 41). However, we observed no significant between-group differences in hemodynamic parameters. In the study, all patients underwent a preoperative paravertebral block, which is well known to inhibit sympathetic nervous system activity. This likely played a major role in the lack of significant differences in MAP and HR between groups. The sympathetic blockade induced by the paravertebral anesthesia may have masked or diminished any additional autonomic effects produced by TEAS, making it more difficult to detect changes in MAP and HR. Therefore, the absence of noticeable differences in MAP and HR does not necessarily exclude a physiological effect of TEAS on thermoregulation.

This study has several limitations. First, core temperature was measured using an infrared tympanic thermometer rather than gold-standard methods; therefore, potential underestimation and misclassification cannot be excluded. Hypothermia was defined as an perioperative temperature <36 °C, regardless of duration. While this approach allows for a more sensitive assessment of whether TEAS reduces the incidence of hypothermia, it does not distinguish between transient and sustained hypothermia, which may have different clinical implications. Consequently, the conclusions should be interpreted with this methodological limitation in mind. Second, the study did not directly assess mechanistic biomarkers, which would have provided more direct evidence supporting the proposed mechanisms. Third, participants may have inferred their group allocation, potentially introducing expectation bias. Although outcome assessors were blinded, treatment providers were not, and the effectiveness of blinding was not formally evaluated, which may have affected internal validity. Finally, forced-air warming was applied only as a rescue intervention rather than as routine prophylactic management, which may limit its applicability in guideline-based clinical settings. Future studies should prioritize multicenter randomized controlled trials with larger sample sizes to validate these findings, explore dose–response relationships for TEAS parameters, and clarify acupoint-specific mechanisms using advanced neuroimaging techniques.

## 5 Conclusion

In conclusion, this study suggests perioperative TEAS as a simple, noninvasive adjunct to improve thermoregulation, as reflected by a reduced incidence of hypothermia and postoperative shivering in patients undergoing VATS. Further multicenter studies are needed to validate these findings and clarify the underlying physiological mechanisms.

## Data Availability

The datasets presented in this article are not readily available because the datasets generated and/or analyzed during the current study are not publicly available due to patient privacy and ethical restrictions but are available from the corresponding author upon reasonable request. Requests to access the datasets should be directed to HL, professorhansh@sina.com.
